# Correlation between Motor Coordination Skills and Emotional and Behavioral Difficulties in Children with and without Developmental Coordination Disorder

**DOI:** 10.3390/ijerph17207362

**Published:** 2020-10-09

**Authors:** Kyujin Lee, Yong Hwan Kim, Yongho Lee

**Affiliations:** 1 Department of Physical Education, Seoul National University, Seoul 08826, Korea; my993286@snu.ac.kr; 2 Institute of Sport Science, Seoul National University, Seoul 08826, Korea; 3 Department of Physical Education, Gangneung-Wonju National University, Gangneung 25457, Korea

**Keywords:** developmental coordination disorder, emotional and behavioral problems, children

## Abstract

The purpose of this study was to compare whole factors of emotional and behavioral problems between children with and without developmental coordination disorder (DCD) and investigate the interrelationship between motor coordination skills and emotional and behavioral problems among the children. As a result of screening participants (288 children) based on DSM-5 standard, participants were classified as DCD and typically developing (TD) groups. A total of 60 children (mean age: 8.8 years ± 3.5 months; DCD group *n* = 30, TD group *n* = 30) were assessed using the Korean Behavior Assessment System for Children, Second Edition for emotional and behavioral problems. Children with DCD showed significantly poor scores in internalizing problems (*p* = 0.009), inattention/hyperactivity (*p* = 0.004), and emotional symptoms index (*p* = 0.001) among the criteria of emotional problems and in personal adjustment (*p* = 0.000) among the criteria of behavioral problems. The MABC-2 composite percentile score of participants showed a significant correlation with internalizing problem behavior (*r* = −0.382, *p* = 0.003), inattention / hyperactivity disorder (*r* = −0.409, *p* = 0.001), emotional symptoms index (*r* = −0.483, *p* = 0.000), and personal adjustment (*r* = 0.474, *p* < 0.01). Our results validated that children with DCD have more emotional and behavioral difficulties than TD children. Our results revealed that the motor coordination skills have correlated with emotional and behavioral difficulties among children.

## 1. Introduction

Approximately 5%–6% of children with symptoms associated with difficulties in performing activities of daily living and the issue of acquiring motor skills due to a lack of coordination rather than a neurological deficit are diagnosed with developmental coordination disorder (DCD) [1,2,3]. Due to the lack fundamental motor skills, children with DCD have difficulty participating in play and sports activities, and thus accumulate negative experiences associated with such activities, eventually leading to the avoidance of such activities [4,5]. This avoidance can have negative effects on cardiovascular-related health outcomes [6], resulting in a higher incidence of being overweight or obese [7], having cardiovascular disease [8], and reduced health-related fitness compared to their peers [9]. Furthermore, DCD may negatively affect aspects of the child’s social inclusion and self-concept formation [10], potentially leading to emotional and behavioral problems [11].

Motor skill impairment dangerously influences psychosocial outcomes, such as anxiety during childhood and adolescence [10,12]. Furthermore, even pre-school age children who lack motor skills feel greater anxiety and depression than same-aged children who are not lacking in motor skills [13]. Problems of children with DCD might continue throughout adolescence and adulthood, causing the increase of various danger factors [14]. Therefore, discovering DCD children’s emotional and behavioral problems in an early stage is very critical for their future development.

Few studies related to emotional and behavioral problems in DCD have reported the differences of emotional and behavioral problems between children with and without DCD. However, it is hard to generalize their results because there were several limitations. Some studies’ participants sampled from clinical environments based on information written by parents and teachers. Other studies obtained data using the unverified methods in children. Furthermore, previous research investigated only a few factors of emotional and behavioral problems.

Therefore, the purpose of this study was to compare whole factors of emotional and behavioral problems between children with and without DCD by using a verified method in children. In addition, the present study investigates the interrelationship between children’s motor coordination skills and emotional and behavioral problem.

## 2. Materials and Methods

### 2.1. Participants

Five elementary schools from the city of Incheon in the South Korea were randomly selected and invited to participate in this study. A total of three schools’ administrators agreed to participate in this project. A total of 288 children (age range of 8–9 years, 148 boys, 140 girls) from the three schools were initially recruited and screened for this research. As a result of screening 288 participants based on the Diagnostic and Statistical Manual of Mental Disorders, Fifth Edition (DSM-5) standards [3], 46 participants (30 boys, 16 girls) were classified as DCD group and 242 participants (118 boys, 124 girls) were classified as typically developing (TD) group. After the DCD screening process we randomly divided the DCD (*n* = 30) and TD group (*n* = 30) in which were placed 15 boys and 15 girls for each group to exclude the influence of gender between groups. A total of 60 children (mean age: 8.8 years ± 3.5 months; DCD 30, TD group 30) were assessed using the Korean Behavior Assessment System for Children, Second Edition (KBASC-2) [15], and the data were used for this study. The characteristics of participants are provided in Table 1. This study was conducted with the approval of the Seoul National University Institutional Review Board (approval No. 1603/001-028).

### 2.2. DCD Screening

We used all four criteria (A: motor coordination skills deficit; B: activities of daily living; C: onset of symptoms; and D: medical condition) defined by the DSM-5. We systematically followed four steps described below based on the previous study [16]. In step 1, as for criteria A, we evaluated if a total score of the Movement Assessment Battery for Children, Second Edition (MABC-2) [17] was below the 15th percentile; in step 2, as for criteria B, we checked if a total score of Developmental Coordination Disorder Questionnaire 2007 [18] was below 55 and if both grades of academic performance and physical education adherence were not above average; in step 3, as for criteria C, we checked student’s age on the school record; in step 4, as for criteria D, we checked students’ health record to confirm if there were any medical conditions or neurological disabilities.

### 2.3. Emotional and Behavioral Problem

Emotional and behavioral problems were evaluated using the KBASC-2, the Korean standardized version of the Behavior Assessment System for Children, Second Edition [19]. KBASC-2 is multi-dimensional mental health screening inspection that provides comprehensive information on the problematic behavior patterns based on the self-perception and attitude toward others of children aged 8–11 years [15]. KBASC-2 provides a validity index, general measure, scale score, measure personality traits, and positive adaptive skills comprehensively in the screening process of emotional symptoms and problematic behaviors and in the evaluation of adaptive resilience, which are useful in evaluating the prognosis in counseling and psychotherapy. KBASC-2 reliability and validity were verified by Ahn, Ebesutain, and Kamphaus [15] and used as a tool for the evaluation of children’s emotions and behaviors in the South Korea elementary school environment.

The KBASC-2 general criterion score is useful to summarize the entire test and to make an extensive conclusion on the various types of personality traits and emotional behavioral problems. The general criteria of KBASC-2 are comprised of school problems, internalizing problems, inattention/hyperactivity, emotional symptoms index, and personal adjustment. Excluding the personal adaptation general criterion evaluating positive adaptation skills, a score of 60–69 is in the range of “high” on the subclinical level, and a score ≥70 is in the range of “very high” on the clinical level. In the personal adaptation general measure, a score of 31–40 is in the range of “low” on the subclinical level, and a score ≤30 is in the range of “very low” on the clinical level.

### 2.4. Statistical Analysis

SPSS (v.25.0 IBM SPSS, New York, NY, USA) was used for analysis. The independent t-test was used to assess the differences between children with DCD and TD children. The correlations between motor coordination skills and emotional and behavioral difficulties were analyzed using Pearson’s correlation coefficient. All data were presented as mean ± standard deviation. Differences were considered significant for *p* < 0.05.

## 3. Results

### 3.1. Difference of Emotional Problems in Children with DCD and TD Children

The differences between children with DCD and TD children’s emotional problems are provided in Table 2. There were statistically significant differences in internalizing problems, inattention/hyperactivity, and the emotional symptoms index but no difference in school problems. Children with DCD showed a significantly high score in internalizing problems (*p* = 0.009), inattention/hyperactivity (*p* = 0.004), and the emotional symptoms index (*p* = 0.001) among the KBASC-2 criteria of emotional problems.

The clinical level, subclinical level, and normal range distribution of participants in emotional problems are shown in Table 3. The distribution of children with DCD who got over the subclinical score in internalizing problems (DCD 4 children 13.3%, TD 0 children 0%), inattention/hyperactivity (DCD 2 children 6.7%, TD 0 children 0%), and the emotional symptoms index (DCD 5 children 16.7%, TD 0 children 0%) higher than TD children. However, the distribution of children with DCD who got over the subclinical score in school problems (DCD 2 children 6.7%, TD 4 children 13.3%) is lower than TD children.

### 3.2. Difference of Behavioral Problem in Children with DCD and TD Children

The differences between children with DCD and TD children’s behavioral problems are provided in Table 4. There were statistically significant differences in personal adjustment. Children with DCD showed a significantly poor score in personal adjustment (*p* = 0.000) among the KBASC-2 criteria of behavioral problem.

The clinical level, subclinical level, and normal range distribution of participants in behavioral problem are shown in Table 5. The distribution of children with DCD who got over the subclinical score in personal adjustment (DCD 7 children 23.3%, TD 0 children 0%) is higher than TD children.

### 3.3. Correlation between the Motor Coordination Skills and Emotional Problems

The correlations between the motor coordination skills and emotional problems are shown in Table 6. The MABC-2 composite percentile score of participants showed a significant correlation with internalizing problem behavior (*r* = −0.382, *p* = 0.003), inattention / hyperactivity disorder (r = −0.409, *p* = 0.001), and emotional symptoms index (*r* = −0.483, *p* = 0.000), which are the emotional problem criteria of the KBASC-2.

### 3.4. Correlation between the Motor Coordination Skills and Behavioral Problem

The correlations between the motor coordination skills and behavioral problems are shown in Table 7. The MABC-2 composite percentile score of participants showed a significant correlation personal adjustment (*r* = 0.474, *p* < 0.01), which are the behavioral problem criteria of the KBASC-2.

## 4. Discussion

Children with DCD have negative experiences of being excluded from sports activities that occupy a significant part of healthy school age development and further intimidate these children [20]. Children with DCD face difficulties with social participation and face teasing and harassment more than TD children because they have characteristics of physical awkwardness or clumsiness [21]. Children with DCD face a decrease in self-worth [10] and difficulty in interpersonal relationships as social isolation deepens [22]. These problems negatively influence children with DCD, leading to them internalizing problems such as depression and anxiety [23]. These internalizing problems that are discovered during childhood tend to continue until adolescence [24].

In this research, children with DCD had a significantly high locus of control, depression, sense of inadequacy, and the subscales of internalizing problems, compared to TD children. Furthermore, although children with DCD do not show a significant difference in social stress and anxiety compared to TD children, a significant difference was discovered in the emotional symptoms index which is composed of social stress, anxiety, and depression. These differences are similar to those seen in research where parents [25,26] and teachers [27] evaluated the psychosocial problems of children with DCD. These differences are also identical to results found in precedent research [23,28] that reported the relationship between motor coordination and depression in school-aged children.

Children with DCD showed a significant difference in relationships with parents, interpersonal relations, self-esteem, self-reliance, and subscales of personal adjustment, compared to TD children in this study. This result shows that 8–9 year-old Korean children with DCD face difficulties with developing amicable relationships, with not only friends at school but also with parents at home, compared to TD children. It also shows that they do not have positive feelings about themselves and have low confidence in their decision-making. These results are in line with precedent research that shows that the motor coordination difficulties of children with DCD cause peer relation difficulties [29,30] and cause not only mental health problems but also emotional problems [10,31] such as depression [14,32] and anxiety [12].

Children with DCD were shown to be at higher risk of not only internalizing problems but also suffering from inattention/ hyperactivity, compared to TD children. This is judged to be because DCD has a coexistence rate of 50% with ADHD and has several common clinical characteristics such as inattention and hyperactivity [33]. The inattention and hyperactivity of children with DCD can be common characteristics with ADHD and can also be distinct characteristics of DCD. Rather, they can be symptoms of the children’s discouragement as a result of a lack of a writing academic achievement, due to a disability in fine motor control [34]. However, these symptoms in children with DCD must be accepted as distinct from children with ADHD because these characteristics do not show in all children with DCD. Moreover, early intervention must be considered for children with DCD who show inattention and hyperactivity because in longitudinal research [14,35,36] that focused on problems in motor development, the authors showed that negative emotions can be caused by inattention, academic problems, etc.

The claim that children with DCD are more at risk than TD children is limited. The measured average score of the children with DCD in the areas of internalizing problems, inattention/hyperactivity, the emotional symptoms index, and the personal adjustment scale are within the normal range that the KBASK-2 provides. However, the DCD group showed a significant difference compared to the TD group in this research. Compared to the TD group, in which there are no children at risk in the subclinical level, there were four children in internalizing problems, two in inattention/hyperactivity, five in the emotional symptoms index, and seven in the personal adjustment scale who were found to be at risk at the subclinical level in the DCD group. Thus, it is judged that children with DCD are at a higher risk in the emotional, behavior field compared to TD children.

The MABC-2 total score showed a significant negative correlation with internalizing problems, inattention/ hyperactivity, the emotional symptoms index, and the composite scales of the KBASC-2 and showed a significant positive correlation with the personal adjustment scale. These results are in line with research that reported the relationship between motor skill impairment and risk factors for poor psychosocial outcomes [10,12]. Regardless of the presence of DCD, emotional behavioral problems decrease as motor coordination ability increases, showing how significant motor coordination is during childhood. Through these results, it has been confirmed that motor coordination ability is a very significant factor in developing relationships with friends at Korean schools. Therefore, the need for school level intervention measures for children who face difficulty with motor coordination should be discussed.

Although some results show that the DCD does not arise from cultural or social environmental influence, the social, cultural environment’s influence during childhood cannot be excluded. Korean society is influenced by Confucianism culture in which women’s participation in sports activities is not looked upon well. Therefore, female children not participating in physical activities is not considered as big of a problem as in male children. In this research, although significant differences of emotional, behavioral problems based on gender were not found, men were evaluated as being at a higher risk compared to women, which was different from the research of Campbell et al. [23]. Furthermore, except for the two female children with DCD who were evaluated to be at a risk level higher than or equal to the subclinical level on the personal adjustment scale, the rest of the children with DCD who were evaluated to be at a risk higher than or equal to the subclinical level were all male children. With only these results, it cannot be concluded that Korean male children with DCD are more highly influenced by emotional social problems compared to female children with DCD. However, the possibility that gender differences exist based on cultural influence can be accepted and additional discussion through further research is required.

This study had some limitations. The present study only recruited children (*n* = 288) from three schools in Incheon city and we assessed the differences between children with DCD and TD children using a small sample size (*n* = 60); therefore, inferences to all Korean children should be made cautiously. We excluded participant with medical diagnosis beside DCD when we screened DCD. It could be a potential bias since the DSM-5 contemplates that DCD can co-occur with other conditions. However, we only excluded one child with intellectual disabilities whose school performance was hard to evaluate for teachers.

## 5. Conclusions

Our study compared the emotional and behavioral difficulties between children with DCD and TD children. In addition, the present study investigated the correlation between motor coordination skills and emotional and behavioral difficulties in children. Our results validated that children with DCD have more emotional and behavioral difficulties than TD children. Our results revealed that the motor coordination skills have correlated with emotional and behavioral difficulties among Korean children using sampling at the public elementary school.

## Figures and Tables

**Table 1:** General characteristics of participants.

	Children with DCD (*n* = 30)	TD Children (*n* = 30)	*p*-Value
Age, Years	8.76 ± 0.29	8.80 ± 0.39	-
Gender, Boys or Girls	15, 15	15, 15	-
Motor Coordination Ability, MABC-2 Score	61.90 ± 5.26	80.71 ± 5.88	<0.001 **

** *p* < 0.01; DCD, developmental coordination disorder; TD, typically developing.

**Table 2:** Difference between children with developmental coordination disorder (DCD) and typically developing (TD) children’s emotional problems.

Scale		Children with DCD (*n* = 30)	TD Children (*n* = 30)	*p*-Value
School Problems	Attitude to School	46.93 ± 9.74	47.50 ± 10.68	0.831
	Attitude to Teacher	46.86 ± 6.18	45.26 ± 7.13	0.357
	(Total) School Problems	46.50 ± 7.37	45.93 ± 9.16	0.793
Internalizing Problems	Atypicality	44.60 ± 3.95	44.60 ± 4.30	1.000
	Locus of Control	48.33 ± 9.19	43.80 ± 5.68	0.025 *
	Social Stress	45.66 ± 7.59	42.83 ± 6.51	0.126
	Anxiety	47.63 ± 6.36	44.73 ± 6.20	0.079
	Depression	51.26 ± 11.94	43.60 ± 5.71	0.002 **
	Sense of Inadequacy	50.46 ± 8.24	44.96 ± 7.24	0.008 **
	(Total)Internalizing Problems	47.86 ± 7.30	43.36 ± 5.54	0.009 **
Inattention/ Hyperactivity	Sense of Inattention	49.86 ± 6.53	43.33 ± 7.79	0.001 **
	Hyperactivity	48.50 ± 5.84	46.50 ± 5.60	0.181
	(Total) Inattention/ Hyperactivity	49.10 ± 5.90	44.46 ± 6.01	0.004 **
Emotional Symptoms Index		49.30 ± 8.72	42.33 ± 6.34	0.001 **

* *p* < 0.05, ** *p* < 0.01; DCD, developmental coordination disorder; TD, typically developing.

**Table 3:** Clinical, subclinical, and normal level distribution of participants in emotional problems.

Scale		Children with DCD (*n* = 30)			TD Children (*n* = 30)		
		Clinical	Subclinical	Normal	Clinical	Subclinical	Normal
School Problems	Attitude to School	2	1	27	2	4	24
	Attitude to Teacher		2	28		2	28
	School Problems	1	1	28	2	2	26
Internalizing Problems	Atypicality			30			30
	Locus of Control	1	2	27			30
	Social Stress		1	29		2	28
	Anxiety		1	29		2	28
	Depression	5	1	24			30
	Sense of Inadequacy		5	25		4	26
	Internalizing Problems		4	26			30
Inattention/ Hyperactivity	Sense of Inattention		1	29		2	28
	Hyperactivity		1	29			30
	Inattention/ Hyperactivity		2	28			30
Emotional Symptoms Index		1	4	25			30

**Table 4:** Difference between children with DCD and TD children’s behavioral problems.

Scale		Children with DCD (*n* = 30)	TD Children (*n* = 30)	*p*-Value
Personal Adjustment	Relations with Parents	51.90 ± 6.62	55.73 ± 7.35	0.038 *
	Interpersonal Relations	50.46 ± 10.86	56.53 ± 3.44	0.005 **
	Self-esteem	50.80 ± 9.02	57.46 ± 5.80	0.001 **
	Self-reliance	47.16 ± 10.96	53.50 ± 8.58	0.016 *
	Personal Adjustment	49.83 ± 9.42	57.93 ± 6.33	0.000 **

* *p* < 0.05, ** *p* < 0.01; DCD, developmental coordination disorder; TD, typically developing.

**Table 5:** Clinical, subclinical, and normal level distribution of participants in behavioral problem.

Scale		Children with DCD (*n* = 30)			TD Children (*n* = 30)		
		Clinical	Subclinical	Normal	Clinical	Subclinical	Normal
Personal Adjustment	Relations with Parents		1	29		1	29
	Interpersonal Relations	1	5	24			30
	Self-esteem	1	5	24			30
	Self-reliance	1	8	21		2	28
	(Total) Personal Adjustment		7	23			30

**Table 6:** The association of motor coordination skills and emotional problems.

Variables		MABC-2 Total Score	*p*-Value
School Problems	Attitude to School	−0.054	0.680
	Attitude to Teacher	−0.180	0.168
	(Total) School Problems	−0.111	0.398
Internalizing Problems	Atypicality	0.091	0.489
	Locus of control	−0.305	0.018 *
	Social Stress	−0.158	0.229
	Anxiety	−0.269	0.038 *
	Depression	−0.468	0.000 **
	Sense of Inadequacy	−0.446	0.000 **
	(Total) Internalizing Problems	−0.382	0.003 **
Inattention/ Hyperactivity	Sense of Inattention	−0.478	0.000 **
	Hyperactivity	−0.171	0.192
	(Total) Inattention/ Hyperactivity	−0.409	0.001 **
Emotional Symptoms Index		−0.483	0.000 **

* *p* < 0.05, ** *p* < 0.01; DCD, developmental coordination disorder; TD, typically developing.

**Table 7:** The association between motor coordination skills and behavioral problems.

Variables		MABC-2 Total Score	*p*-Value
Personal Adjustment	Relations with Parents	0.282	0.029 *
	Interpersonal Relations	0.349	0.006 **
	Self-esteem	0.360	0.005 **
	Self-reliance	0.409	0.001 **
	(Total) Personal Adjustment	0.474	0.000 **

* *p* < 0.05, ** *p* < 0.01; DCD, developmental coordination disorder; TD, typically developing.
